# Delayed Presentation of Suture Erosion following Burch Colposuspension

**DOI:** 10.1155/2017/8178361

**Published:** 2017-07-13

**Authors:** Robert Shapiro, Ali Hajiran, Stanley Zaslau

**Affiliations:** Department of Obstetrics/Gynecology, West Virginia University, Morgantown, WV, USA

## Abstract

**Background:**

Synthetic mid-urethral mesh slings are the most common primary surgical treatment for stress urinary incontinence (SUI) and have been designated as the standard of care by the American Urogynecologic Society. In recent years, synthetic mesh has come under increased scrutiny by the Federal Drug Administration (FDA) due to concerns over patient safety. This has led to more surgeons and patients preferring Burch colposuspension to treat SUI.

**Case:**

We discuss two cases of suture erosion into the urethra and bladder. They presented with irritative voiding symptoms and recurrent urinary tract infections. Both were discovered years after a Burch colposuspension.

**Conclusion:**

As reported in the literature as early as 1999, erosion is a complication associated with many types of incontinence surgery and not unique to mesh based sling operations. Burch colposuspension should not be favored solely to avoid erosion and patients should be counseled accordingly.

**Teaching Point:**

Cystourethroscopy performed intraoperatively or postoperatively is essential for early diagnosis and treatment of complications related to incontinence surgery.

## 1. Introduction

Stress urinary incontinence (SUI) is defined as the involuntary loss of urine with provocative maneuvers, such as cough, laugh, and sneeze [1]. Synthetic mid-urethral mesh slings are the most common primary surgical treatment for SUI and have been designated as the standard of care by the American Urogynecologic Society [2]. In recent years, synthetic mesh has come under increased scrutiny by the Federal Drug Administration (FDA) due to concerns over patient safety [3].

A major complication of mid-urethral slings is erosion where tissue overlying the mesh becomes thin and weak leading to exposure within an adjacent organ [4]. Vaginal erosion rates for mesh repairs range from 7% to 20% and may present several years after the index procedure [5]. Burch procedures are being performed with greater frequency in recent years, in part due to the FDA notification about synthetic mesh use [6].

The complication of erosion is not unique to sling operations. This can also occur with Burch procedures. We present 2 cases of suture erosion into the urethra and bladder, both discovered years after a Burch colposuspension.

## 2. Case Presentation

### 2.1. Case 1

A 67-year-old white female with urinary hesitancy, frequency, and urgency incontinence was referred to our clinic for urogynecology consultation after failing standard therapy with approximately 6 months of anticholinergic medications and behavioral modifications. Past surgical history was significant for a Burch colposuspension about 10 years prior to examination. Past medical history was significant for chronic obstructive pulmonary disease having been a prior smoker. She quit approximately 5 years prior to evaluation. Physical examination revealed a Grade 3 rectocele and Grade 3 enterocele. Cough stress test in lithotomy was negative. Urethral hypermobility was less than 30 degrees. Urodynamics confirmed elevated bladder pressures and increased bladder sensation which was consistent with obstructed voiding.

**Figure 1:**
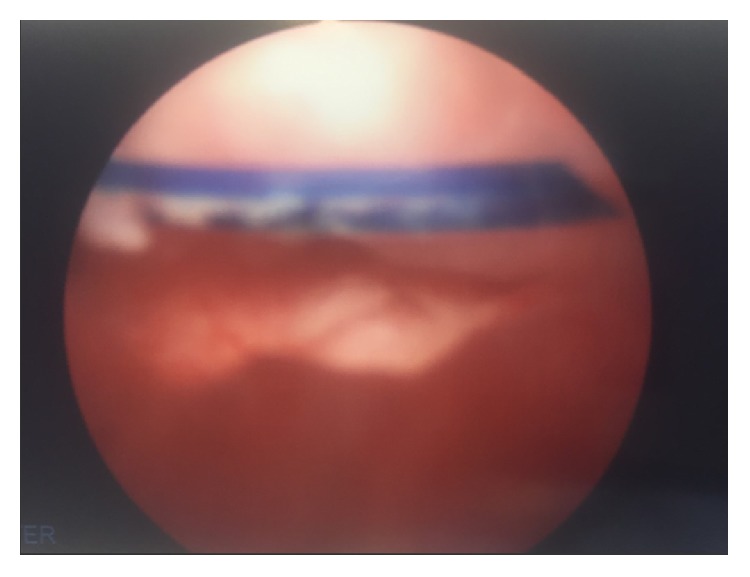
Cystoscopic image of an eroded suture at the level of the proximal urethra near the bladder neck.

Cystourethroscopy was performed which showed an eroded suture at the level of the proximal urethra near the bladder neck (Figure 1). We utilized a 24 Fr resectoscope with a cold-knife to cut the suture. The suture retracted back into the bladder and was removed with cystoscopy at the end of the case.

The prolapse and obstructed voiding was treated with abdominal sacrocolpopexy. Her postoperative course was uneventful and the patient had complete resolution of symptoms by her 6-week postoperative visit.

### 2.2. Case 2

A 48-year-old white female with recurrent urinary tract infections for the past year was referred to our clinic for urogynecology consultation having failed treatment with nitrofurantoin suppression. Past surgical history was significant for a Burch colposuspension six years earlier. Past medical history was significant for chronic obstructive pulmonary disease and chronic bronchitis. The patient had a 30-pack year smoking habit. Physical examination revealed no significant pelvic prolapse. Cough stress test in lithotomy was negative. Urethral hypermobility was less than 30 degrees.

**Figure 2:**
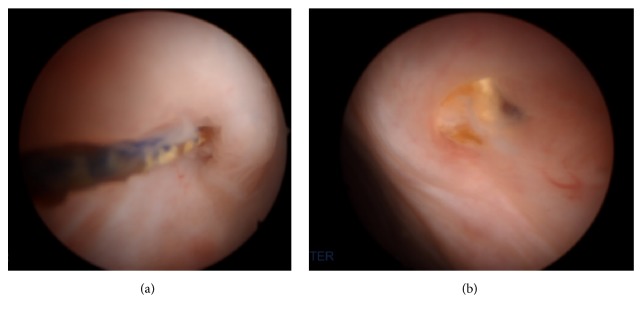
Cystoscopic images showing suture erosion at the right and left anterior bladder wall just inferior to the dome.

Cystourethroscopy was performed which showed suture erosion at the right and left anterior bladder wall just inferior to the dome (Figures 2(a) and 2(b)).

The patient underwent laser cystolitholapaxy to remove the eroded suture material. Her postoperative course was uneventful. The patient presented for a 3-month follow-up without a recurrence of urinary tract infection.

## 3. Discussion

Highly publicized medicolegal trials in the US imply that some of the worst complications surrounding incontinence surgery are unique to mesh. This has led to more surgeons and patients preferring abdominal colposuspension to treat SUI. Our cases illustrate that suture injury/erosion is not unique to synthetic mid-urethral sling operations.

In the first case, the suture erosion was most likely a source of bladder irritation contributing to the patient's urgency and incontinence. This type of complication related to Burch is well described. In as early as 1999, Dwyer et al. reported suture misplacement or erosion as an infrequent but important complication of Burch colposuspension and should be suspected with persistent irritative voiding symptoms [7].

Vaginal enterocele, as seen in this patient, occurs in up to 40 percent of cases after Burch [8]. Overelevation of the bladder neck at the time of Burch can change the vesicovaginal angle resulting in this type of pelvic prolapse. Treating the enterocele with sacrocolpopexy and removing the extruded suture via cystoscope was done to avoid reopening the space of Retzius. Significant scar tissue is likely to be encountered with reopening the space of Retzius, thereby increasing the risk of hemorrhage and bladder injury. Similarly, laser cystolitholapaxy was used to treat the eroded suture material in the second case to avoid the space of Retzius and decrease the risk of significant patient morbidity.

In conclusion, Burch colposuspension is thought to carry a lesser risk of complications of erosion of a foreign body and is thus being favored by many patients and physicians over synthetic slings for the treatment of SUI. However, as we have demonstrated with these two case reports, suture erosion can occur following Burch and may present in a delayed fashion. A high index of suspicion may be required to make the diagnosis given the variable symptom presentation. Cystoscopy is critical to diagnosis.

## 4. Conclusions

1. Erosion is a complication associated with different types of incontinence surgery and not unique to mesh based sling operations.
2. Burch colposuspension should not be favored solely to avoid erosion, and patients should be counseled accordingly.
3. Cystourethroscopy performed intraoperatively or postoperatively is essential for early diagnosis and treatment of complications related to incontinence surgery.
